# Time variation in the probability of failing to detect a case of polymerase chain reaction testing for SARS-CoV-2 as estimated from a viral dynamics model

**DOI:** 10.1098/rsif.2020.0947

**Published:** 2021-04-21

**Authors:** Keisuke Ejima, Kwang Su Kim, Shoya Iwanami, Yasuhisa Fujita, Ming Li, Roger S. Zoh, Kazuyuki Aihara, Taiga Miyazaki, Takaji Wakita, Shingo Iwami

**Affiliations:** ^1^ Department of Epidemiology and Biostatistics, Indiana University School of Public Health-Bloomington, Bloomington, IN 47405, USA; ^2^ Department of Biology, Faculty of Sciences, Kyushu University, 744 Motooka Nishi-ku, Fukuoka 819-0395, Japan; ^3^ International Research Center for Neurointelligence, The University of Tokyo Institutes for Advanced Study, The University of Tokyo, Tokyo, Japan; ^4^ Department of Infectious Diseases, Nagasaki University Graduate School of Biomedical Sciences, Nagasaki, Japan; ^5^ Department of Virology II, National Institute of Infectious Diseases, Tokyo, Japan; ^6^ MIRAI, JST, Saitama, Japan; ^7^ Institute for the Advanced Study of Human Biology (ASHBi), Kyoto University, Kyoto, Japan; ^8^ NEXT-Ganken Program, Japanese Foundation for Cancer Research (JFCR), Tokyo, Japan; ^9^ Science Groove Inc., Fukuoka, Japan

**Keywords:** COVID-19, false-negative rate, PCR test, virus dynamics

## Abstract

Viral tests including polymerase chain reaction (PCR) tests are recommended to diagnose COVID-19 infection during the acute phase of infection. A test should have high sensitivity; however, the sensitivity of the PCR test is highly influenced by viral load, which changes over time. Because it is difficult to collect data before the onset of symptoms, the current literature on the sensitivity of the PCR test before symptom onset is limited. In this study, we used a viral dynamics model to track the probability of failing to detect a case of PCR testing over time, including the presymptomatic period. The model was parametrized by using longitudinal viral load data collected from 30 hospitalized patients. The probability of failing to detect a case decreased toward symptom onset, and the lowest probability was observed 2 days after symptom onset and increased afterwards. The probability on the day of symptom onset was 1.0% (95% CI: 0.5 to 1.9) and that 2 days before symptom onset was 60.2% (95% CI: 57.1 to 63.2). Our study suggests that the diagnosis of COVID-19 by PCR testing should be done carefully, especially when the test is performed before or way after symptom onset. Further study is needed of patient groups with potentially different viral dynamics, such as asymptomatic cases.

## 1. Introduction

In persons with signs or symptoms consistent with COVID-19, or with a high likelihood of exposure (e.g. history of close contact with a confirmed case, travel history to an epicentre), viral testing combined with other tests (e.g. X-ray) is recommended for the diagnosis of acute infection [1]. Viral tests (such as the polymerase chain reaction (PCR) test) look for the presence of SARS-CoV-2, the causative virus of COVID-19. Viral testing is also recommended to screen asymptomatic individuals regardless of suspected exposure to the virus for early identification and to survey the prevalence of infection and disease trends [1]. Although antibody testing is another option to confirm infection, it is used to confirm past infection, because it takes a few weeks for antibody levels to reach detectable amounts after infection [1,2].

PCR tests for SARS-CoV-2 vary according to the sampling process used (i.e. sampled by patients or by healthcare workers [3]), the specimen type (upper and lower respiratory tract, saliva, blood, stool [4,5]), the collection kit, and different target and detection limits [6–8]. Further, test results can differ among runs, laboratories and PCR assays. It is still under debate which specimen type is best. The choice of specimen type should be determined by the quality of the test (i.e. sensitivity and specificity) and by the safety and purpose of the test. For example, saliva samples can be self-collected, which will mitigate the risk of infection of healthcare workers and which is helpful for mass screening [9–12]. However, saliva samples from some patients can be thick, stringy and difficult to pipette [13]. Meanwhile, the viral load in nasal samples collected by patients was reported to be not as high as that in nasopharyngeal swabs collected by health practitioners, which yields lower sensitivity of nasal samples collected by patients [3].

In the context of controlling the COVID-19 pandemic, the probability of failing to detect a case appears to be the most important metric. Note that the probability is not the same as the false-negative rate. The false-negative rate is the probability of negative results given that a swab contains viral genetic material, whereas the probability of failing to detect a case is the probability that an individual is infected (and potentially infectious) but the sample provided is like to not have any viral material in it due to either being prior to viral shedding or at a stage of infection where viral load is below the threshold of consistent detection. Indeed, failing to detect a case leads to lifting precautions and isolation for patients who are still infectious, thus further increasing the transmission risk in households and communities. In contrast, the probability of falsely detecting a case is considered negligible in general unless there are technical errors or contamination in the reagent [2].

The sensitivity of a PCR test is influenced by the sampling process and other factors including the quality of sample collection [14]. Although not frequently discussed, sensitivity is also dependent on the timing of sample collection [2]. Viral load typically increases exponentially during the acute phase of infection, hits a peak, and then declines and disappears. Because sensitivity is dependent on the viral load, the probability of failing to detect a case changes corresponding to the temporal dynamics of viral load. In particular, the probability of failing to detect a case is high at the beginning of infection and long after infection, and it is low when the viral load hits its peak. For example, Kucirka *et al*. [15] and Borremans *et al*. [4] showed that the probability of failing to detect a case of SARS-CoV-2 tests varies dependent on time since exposure or onset. The lowest rate was achieved 3 days after symptom onset, which corresponds to peak viral load as observed in clinical data and as estimated from mathematical models [16–21]. However, most of the data used by Kucirka *et al*. and Borremans *et al*. were collected after symptom onset. Furthermore, those authors estimated the probability of failing to detect a case before symptom onset by using data from a single person, which may be an extremely poor estimate of the true probability of failing to detect a case. Kucirka also did not consider the different types of tests used in the different studies, which is problematic because the detection limit varied between studies.

In the present study, we investigated the probability of failing to detect a case over time by using a viral dynamics model rather than observed test results. Our approach enabled us to investigate the probability of failing to detect a case even before symptom onset by extrapolating the viral load before symptom onset from the model and allowed us to derive the probability of failing to detect a case for different detection limits. First, we parametrized the viral dynamics model by fitting the model to the data. Then, we ran simulations based on the parametrized viral dynamics model, adding errors to create realistic viral-load distributions, and computed the probability of failing to detect a case over time.

## 2. Results

### 2.1. Simulation to compute the probability of failing to detect a case over time

**Figure 1.**
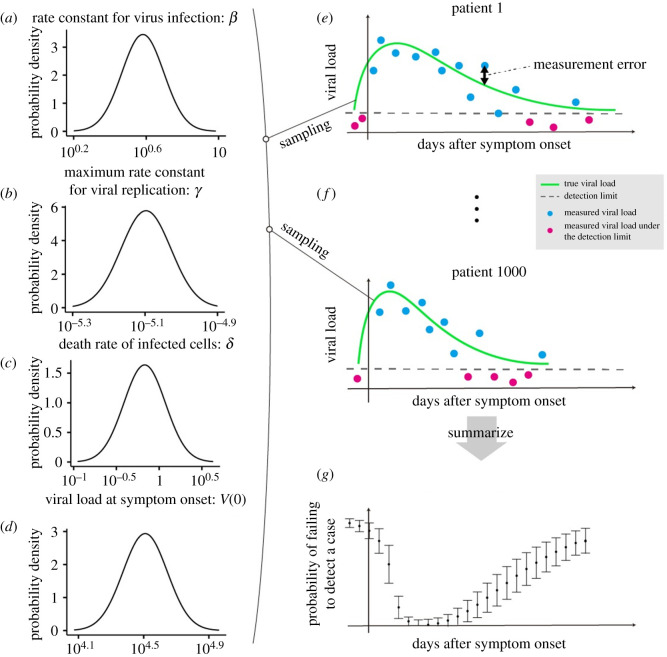
Process of computing the probability of failing to detect a case. The parameter distributions are estimated by fitting the viral dynamics model to the viral load data extracted from clinical studies of SARS-CoV-2 (*a–d*). The parameter values are resampled from the estimated parameter distributions, and 1000 expected viral loads are computed by running the viral dynamics model (*e*,*f*). The green lines correspond to the computed expected viral load. The dashed grey line is the detection limit. The red and blue dots are the measured viral load, which is calculated by adding measurement error to the expected viral load. Then, we calculated the probability of failing to detect a case on day *t*, which is the number of negative measurements (red dots) among all the measurements on day *t* (*g*).

Using the parametrized viral dynamics model, we computed the viral-load distribution over time with days since symptom onset as the time scale. The fitted viral dynamics and the data are depicted in the electronic supplementary material, figure S1 and table S1. We randomly resampled the parameter set (i.e. *β*, *γ*, *δ* and *V*(0)) from the estimated distributions (lognormal distributions), accounting for both fixed-effect estimation and variation in random effects, and ran the model (see Methods). We assumed that the viral load obtained by running the model is *expected viral load*. Thus, each viral load curve corresponds to each patient; in other words, the parameter distributions reflect a random-effect component that accounts for individual variability. However, what we obtain from the PCR test is subject to some measurement error. Thus, we added the measurement error to the expected viral load to obtain *measured viral load* data. We assumed that the error follows a normal distribution with a mean of zero and the variance on log 10 transformed viral load, computed in the process of fitting. In other words, we assumed that the error is independent and identically distributed (i.e. the errors are not correlated between patients or within patients from multiple measurements). We repeated this process 1000 times to create the viral-load distribution over time. The probability of failing to detect a case is computed as the proportion of cases with a viral load below the detection limit at day *t* (*t* ∈ { − 2 , … , 20}), denoted by *p*(*t*): $p(t)=\sum_{i=1}^{1000}I(\mathrm{VL}{\left(t\right)}_{i}<\mathrm{DL})/1000$, where VL(*t*)*_i_* is the measured viral load of individual *i* at time *t*, DL is the detection limit and *I* is the identity function. The large-sample 95% confidence intervals (CIs) of the probability of failing to detect a case were computed by assuming a binominal distribution: $p(t)\pm 1.96\sqrt{\;p(t)(1-p(t))/1000}$. Note that the detection limit varied depending on the test assay [7,22,23]. The lowest was 1 copy ml^−1^ and the highest was over 1000 copies ml^−1^. We used 100 copies ml^−1^ because it is roughly the median value that we have seen in the literature. As a sensitivity analysis, we performed the same simulation using different detection limits (10 and 1000 copies ml^−1^). The computational process is summarized in figure 1.

### 2.2. Time-dependent probability of failing to detect a case during SARS-CoV-2 infection

**Figure 2.**
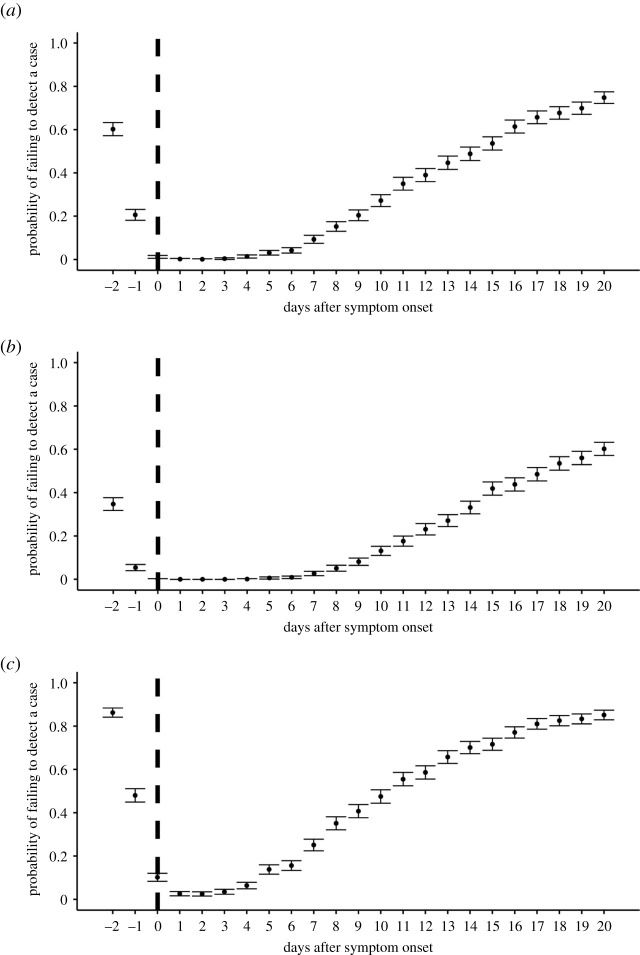
The probability of failing to detect a case over time with different DLs. (*a*) DL = 100 copies ml^−1^, (*b*) DL = 10 copies ml^−1^, (*c*) DL = 1000 copies ml^−1^. The dots are the estimated probability of failing to detect a case at each time point and the bars correspond to the 95%CIs. The vertical dashed lines show the day of symptom onset.

Figure 2*a* shows the computed probability of failing to detect a case over time with a detection limit of 100 copies ml^−1^. As expected from typical viral dynamics, the probability of failing to detect a case was high during the early phase of infection because of the low viral load, which is consistent with previous studies [4,15]. Before symptom onset, the probability of failing to detect a case was over 20% (60.2% (95%CI: 57.2% to 63.2%) at 2 days before symptom onset), suggesting it is difficult to identify all presymptomatic cases with viral testing. The probability of failing to detect a case is minimized at 2 days after symptom onset: 0.1% (95%CI: 0% to 0.3%), which corresponds to the timing of peak viral load. After that, the probability of failing to detect a case increases as the viral load declines or as a virus is eliminated from patients. As a sensitivity analysis, we also computed the probability of failing to detect a case for different detection limits (figure 2*b,c*: detection limit = 10 copies ml^−1^ and 1000 copies ml^−1^, respectively) and confirmed similar trends. The probability of failing to detect a case was high with a higher detection limit: the rate was over 40% before symptom onset with the detection limit of 1000 copies ml^−1^.

## 3. Discussion

We computed the probability of failing to detect a case of PCR test over time using a viral dynamics model. The probability of failing to detect a case was substantially high (over 20%) before symptom onset. The lowest probability of failing to detect a case appeared 2 days after symptom onset. After that, the probability of failing to detect a case declined as the virus was gradually washed out from the host. A similar time trend was observed for different detection limits; however, a higher detection limit yielded a higher probability of failing to detect a case.

We need to be careful in interpreting the probability of failing to detect a case before symptom onset as computed based on our approach. We simply hindcasted the model without considering the timing of infection. Therefore, the viral load we computed may not exist if it is before infection. This becomes a serious issue when we compute the probability of failing to detect a case way before symptom onset. For this reason, we decided to show the probability of failing to detect a case from 2 days before symptom onset, because the 2.5%ile of the incubation period was 2.2 days [15]. In other words, most of the simulated patients are infected and shedding virus 2.2 days before symptom onset. Further study may be needed to consider the timing of infection for a more accurate estimation of the probability of failing to detect a case.

Providing an accurate probability of failing to detect a case is of importance in understanding the epidemiology of COVID-19 as well as its clinical characteristics. For example, the detected prevalence of COVID-19 in the general population based on PCR testing was recently reported from England [24]. The data provide a baseline for monitoring prevalence prospectively and will be useful in, for example, assessing the impact of countermeasures against the COVID-19 pandemic. However, the detected prevalence could be influenced by the probability of failing to detect a case. Given that the probability of failing to detect a case is dependent on the time of specimen collection, recording the timing of the test (days since symptom onset) might be helpful in estimating the true prevalence by accounting for the probability of failing to detect a case. Our estimated probability of failing to detect a case before symptom onset is also suggestive for contact tracing or quarantine, in which cases before symptom onset would be tested; we do not recommend using PCR testing to rule out infected cases in those situations. Further, we do not recommend fully depending on the results of the PCR test in diagnosis, given its non-negligible probability of failing to detect a case depending on the timing of the test. Comprehensive medical tests such as chest X-ray and interviewing for contact history would complement the PCR test for acute cases.

PCR tests have been extensively used in SARS-CoV-2 research because of their high sensitivity and specificity compared with other tests such as antibody and antigen tests. However, this does not undermine the value of other tests, and appropriate tests should be chosen depending not only on their sensitivity and specificity but also on the purposes of testing and the cost [25–27]. For example, frequency has been suggested to be more important than sensitivity for screening purposes [27]. For influenza, rapid molecular assays (i.e. nucleic acid amplification tests) and rapid influenza diagnostic tests (RIDTs) have been extensively used for diagnosis purposes for outpatients [28]. A meta-analysis reported the sensitivity of the RIDT to be 62.3%, which was assessed by using the PCR test as a gold standard (thus 100% sensitivity is assumed for the PCR test) [29]. The sensitivity peaks around 2 to 3 days after symptom onset [29,30], which corresponds to the viral load peak [31] and is in line with our finding for SARS-CoV-2.

The strength of our approach is that we used viral dynamics rather than the observed probability of failing to detect a case, which enabled us to assess the probability of failing to detect a case at time points for which available data were scarce, especially before symptom onset. One of the reasons for the limited data before symptom onset is that people are rarely tested before symptom onset, as the test is more commonly used for diagnosis rather than for screening or surveillance. Although Kucirka *et al*. and Zhen *et al*. estimated the probability of failing to detect a case over time using observed test results, the estimation for before symptom onset was dependent on a single set of data, which we do not believe is a reliable estimation [15,32]. Another strength of our approach is that we can estimate the probability of failing to detect a case for different detection limits because we estimated the distribution of viral load at each time point. Further, although we specifically computed the probability of failing to detect a case for SARS-CoV-2, the framework is applicable to other viruses causing acute respiratory infection, including influenza.

A few points need to be addressed in future studies. We used the viral load measured in upper respiratory specimens because such specimens are prevalently used for the PCR test. However, using saliva may also be considered because the collection of saliva specimens is easy and safe for healthcare practitioners, and the viral load is high enough compared with that from nasopharyngeal specimens, which is a gold standard approach [5,9,11,33]. It might be worth computing the probability of failing to detect a case for saliva specimens if the viral dynamics are not the same as in upper respiratory specimens. Further, the probability of failing to detect a case might be computed for subgroups of the population. In our previous study, we found that viral load dynamics is highly variable among cases [17]. Virus shedding continued for 10 days after symptom onset in some patients but continued for more than 30 days after symptom onset in others. Therefore, the probability of failing to detect a case should differ between those patient groups. If any biomarkers or demographics (i.e. age, sex, race/ethnicity) differentiating the viral dynamics are identified, they should be considered in computing the probability of failing to detect a case. We used only symptomatic cases in this study because data from asymptomatic cases were not available. However, the probability of failing to detect a case could differ between symptomatic and asymptomatic cases. Although the difference in duration of virus shedding between symptomatic and asymptomatic cases is still controversial from the literature [34,35], viral load dynamics and the corresponding probability of failing to detect a case might be dependent on the presence or absence of symptoms. Similarly, the probability of failing to detect a case was nearly zero in the first week since symptom onset, which might be because the data included only hospitalized patients. Indeed, the viral load is known to be positively associated with disease severity [36–38]. Lastly, we need to update the viral dynamics model accounting for new findings once available. For example, if a complex immunologic response is important and measured over time, such mechanisms should be incorporated in the model. As such data are still limited, we used the simplest model.

We computed the probability of failing to detect a case of the PCR test over the time course of infection using a viral dynamics model. The computed probability of failing to detect a case needs to be considered in the context of catching cases (such as screening, test and trace, and epidemiological surveillance).

## 4. Methods

### 4.1. Data

The longitudinal viral load data were extracted from four COVID-19 clinical studies [21,39–41]. The data include only symptomatic and hospitalized cases. The viral load was measured continuously within the interval of a few days since hospitalization. For some studies, the viral load was measured from different specimens (i.e. sputum, stool, blood); however, we used the data from upper respiratory specimens because (1) the upper respiratory tract is the primary target of infection, (2) these specimens are commonly used for diagnosis and (3) for consistency of the data. Data from patients under antiviral treatment and data with less than two data points were excluded from the analysis. Ethics approval was obtained from the ethics committee of each medical/research institute for each study. Written informed consent was obtained from patients or their next of kin, as was described in the original papers. We summarize the data in table 1.

**Table 1.** Summary of data.

papers	country	no. of included (excluded) cases	site of viral load data used	reporting value	detection limit (copies ml^–1^)	range of symptom onset	age^c^	sex (M : F)
Young *et al.* [40]	Singapore	12 (6)	nasopharyngeal swab	cycle threshold^a^	68.0	1/21–1/30	37.5 (31–56)	6 : 6
Zou *et al.* [39]	China	8 (8)	nasal swab	cycle threshold^a^	15.3	1/11–1/26	52.5 (28–78)	3 : 5
Kim *et al.* [41]	Korea	2 (7)	nasopharyngeal and oropharyngeal swab	cycle threshold^a^	68.0	NA	NA	NA
Wölfel *et al.* [21]	Germany	8 (1)	pharyngeal swab	viral load (copies/swab)^b^	33.3	1/23–2/4	NA	NA

^a^cycle threshold values were converted by using the formula: log_10_(viral load [copies ml^−1^]) = −0.32 × Ct values [cycles] + 14.11 [39].

^b^1 swab was assumed to be 3 ml in Wölfel *et al.* according to the original paper.

^c^median (range).

### 4.2. A mathematical model for virus dynamics and parameter estimation by nonlinear mixed-effect model

Following is the mathematical model describing viral dynamics, previously proposed in [18,42,43]:

$$\begin{array}{rl} \frac{df(t)}{dt}= & -\beta f(t)V(t)\\ \;\mathrm{and}\;\;\;\;\frac{dV(t)}{dt}= & \gamma f(t)V(t)-\delta V(t), \end{array}$$

where *f*(*t*) is the relative fraction of uninfected target cell population at day *t* to that at day 0 (i.e. *f*(0) = 1), and *V*(*t*) is the amount of virus at day *t*. This two-dimensional model was derived from the three-dimensional model composed of viruses, uninfected cells and infected cells by assuming a quasi-steady state of the number of viruses [42]. This assumption is reasonable for most of the viruses causing acute infectious disease because the clearance rate of the virus is typically much larger than the death rate of the infected cells as evidenced *in vivo* [42,44,45]. Note that time 0 corresponds to the day of symptom onset for practical purposes. The parameters *β*, *γ* and *δ* are the rate constant for virus infection, the maximum rate constant for viral replication and the death rate of infected cells, respectively. The viral load data from the five different papers were fitted using a nonlinear mixed-effect model accounting for inter-individual variability in each parameter. Specifically, the parameter for individual *k* is presented by $\theta \times e^{\pi_{k}}$, where *θ* is the fixed effect and *π_k_* is the random effect, which follows the normal distribution: *N*(0, *σ*). Fixed-effect parameters and random-effect parameters were estimated using the stochastic approximation EM (expectation-maximization) algorithm and empirical Bayes' method, respectively. The mixed model approach is commonly used to analyse longitudinal viral load data [46,47], because the model can account for variability in parameters between cases, and parameter estimation is feasible for cases with limited data points. We used MONOLIX 2019R2 for the implementation of parameter estimation [48]. To account for data points under detection limits (the detection limits were 15.3 copies ml^−1^ [39], 33.3 copies [21] and 68 copies ml^−1^ [40,41], respectively), the likelihood function assumed that data under the detection limit are censored [49]. Finally, we fitted the normal distribution with mean zero to the difference between the model and empirical viral load data to estimate the variance of the error.

## Data accessibility

The codes and data used in this paper are available from the corresponding authors on reasonable request.
